# *Mycoplasma pneumoniae* Mediastinal Lymphadenitis in Children: A Case Series and a Review of the Literature

**DOI:** 10.1097/INF.0000000000004578

**Published:** 2024-10-10

**Authors:** Laura Martino, Cristina De Rose, Rosa Morello, Ilaria Lazzareschi, Francesco Proli, Giulia Bersani, Marilena La Sorda, Piero Valentini, Danilo Buonsenso

**Affiliations:** * Department of Woman and Child Health and Public Health, Fondazione Policlinico Universitario A. Gemelli IRCCS, Rome, Italy; † Department of Laboratory and Infectious Sciences, Fondazione Policlinico Universitario A. Gemelli IRCCS, Rome, Italy; ‡ Global Health Research Institute, Istituto di Igiene, Università Cattolica del Sacro Cuore, Rome, Italy, Area Pediatrica, Dipartimento di Scienze della Vita e Sanità Pubblica, Università Cattolica del Sacro Cuore, Rome, Italy

## To the Editors:

*Mycoplasma pneumoniae* is a common cause of upper and lower respiratory tract infections in children. The most frequent clinical manifestations include pharyngotonsillitis, acute bronchitis and community-acquired pneumonia, which is usually mild and self-limited while it can be severe and complicated in patients with immunodeficiency and chronic cardiorespiratory diseases. *Mycoplasma pneumoniae* infection accounts for 10%–40% of community-acquired pneumonia in children between 9 and 14 years old, and it is less frequent before 5 years of age.^1,2^

*Mycoplasma pneumoniae* infection can also be responsible for rare extrapulmonary manifestations.^3,4^ Macrolides (azithromycin, clarithromycin, erythromycin) are the recommended first-line treatment for moderate to severe *Mycoplasma pneumoniae* infection in school-age children.^5,6^ However, the efficacy and clinical benefit of antibiotic therapy for this kind of infection is controversial.

In this article, we report the cases of 2 children with *Mycoplasma pneumoniae* infection detected through polymerase chain reaction on nasopharyngeal swab who were admitted to our emergency department for persistent fever and had radiologic evidence of parenchymal hypoventilation and severe mediastinal lymphadenitis suspicious of active pulmonary tuberculosis. In addition, we performed a review literature to better investigate the association between *Mycoplasma pneumoniae* and mediastinal lymphadenitis.

## CASE 1

A 12-year-old girl with no significant medical history was admitted to our emergency department for fever for 4 days and chest pain. She came back from Romania the month before the appearance of clinical manifestations, where she met her grandfather who was receiving antitubercular treatment. The patient had just completed a 3-day treatment with clarithromycin prescribed by her pediatrician without clinical benefit. She had not experienced increased work of breathing and her vital parameters were the following: temperature 37.7°C, blood pressure, heart rate, respiratory rate and peripheral oxygen saturation were normal. The physical examination showed bilateral basal hypophonesis in the respiratory auscultation, with abolished vesicular murmur, no wheezing.

**FIGURE 1.**
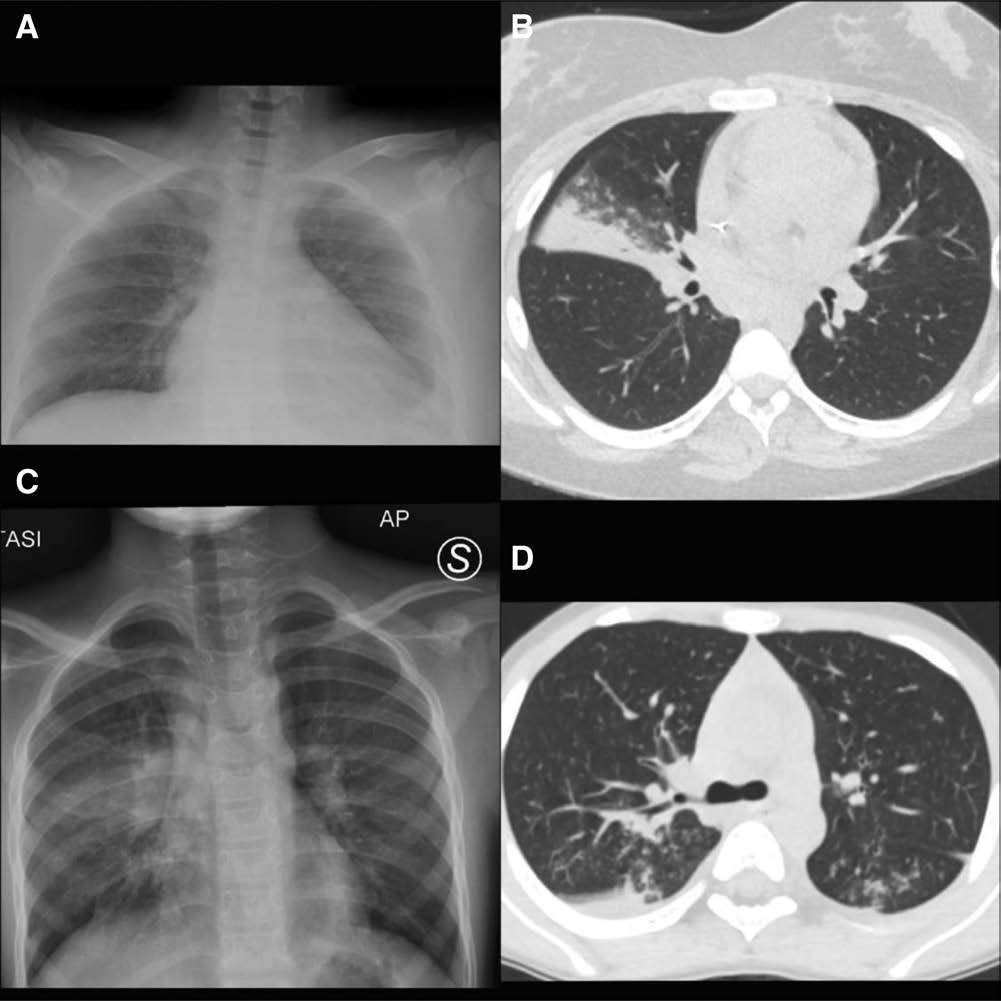
Radiologic findings in 2 patients. A: Chest radiograph showing left pleural effusion associated with basal consensual parenchymal hypoventilation; (B) chest CT scan revealing a ribbon-like area of parenchymal consolidation with air bronchogram. This formation is surrounded by several areas of slightly increased density of the pulmonary parenchyma, with ground-glass appearance, with multiple centrilobular micronodules some of which had a tree-in-bud aspect. In the right superior lobe, some calcified nodular formations are described, associated with calcified lymph nodes, compatible with the primary complex. Furthermore, there are several mediastinal lymphadenopathies. C: Chest radiograph showing a right hilar round-shaped parenchymal hypoventilation, surrounded by another medio-basal hypoventilation; (D) Chest CT scan revealing several bilateral areas of parenchymal consolidation, with irregular margins and a confluent appearance, and partially obstructed bronchi; several mediastinal lymphadenopathies, some of which characterized by internal necrosis. CT indicates computed tomography.

Blood laboratory tests revealed a C-reactive protein of 173.5 mg/L [normal values (n.v.) 0.00–5.00 mg/L], white blood cells 15.400/mmc (n.v 4.5–13.000/mmc), neutrophils 11.110/mmc (n.v. 1.5–6.000/mmc), normal hepato-renal function. Chest radiograph (Fig. 1A) was required and it showed left pleural effusion associated with basal consensual parenchymal hypoventilation and a bit enlarged cardiac shape.

The patient was started on amoxicillin and clavulanic acid endovenous treatment and was admitted to the pediatric ward. We performed a bedside lung ultrasound showing bilateral pleural effusion and several atelectasis, so the antibiotic treatment was shifted to ceftriaxone. Since we did not observe clinical benefit, we added vancomycin to the treatment. In addition, due to the provenience from a high-tuberculosis (TB) burden country, we performed Mantoux intradermal skin test which resulted positive. Quantiferon test and microbiologic diagnostic tests to find *Mycobacterium tuberculosis* on 3 samples of sputum were negative.

A chest computed tomography (CT) scan (Fig. 1B) was required to better characterize the images revealed with lung ultrasound and it showed a ribbon-like area of parenchymal consolidation with air bronchogram. This formation was surrounded by several areas of slightly increased density of the pulmonary parenchyma, with ground-glass appearance, with multiple centrilobular micronodules some of which had a tree-in-bud aspect. In the right superior lobe, some calcified nodular formations were described, associated with calcified lymph nodes, compatible with the primary complex. Interestingly, the CT scan showed several reactive mediastinal lymphadenopathies. In suspicion of active pulmonary tuberculosis, specific first-line antitubercular treatment was started.

Because of the persistence of fever 15 days after the admission and the negativity of microbiologic analysis made on sputum, a fibrobronchoscopy was performed to obtain bronchoalveolar lavage fluid and to do a needle aspiration of the subcarinal lymphadenopathies. *Mycoplasma pneumoniae* was found both on nasopharyngeal swab and bronchoalveolar lavage fluid. The patient underwent a new chest CT scan 3 weeks later showing a significant reduction of the areas of parenchymal consolidation, whereas the mediastinal lymphadenitis was not resolved at all. In consideration of negativity of microbiologic examinations, during a follow-up evaluation, the antitubercular treatment was interrupted. Long-term follow-up confirmed full recovery and no new diagnosis of TB.

## CASE 2

A 9-year-old boy with no significant medical history was referred to our emergency department for fever and productive cough for 10 days. He was originally from India and his father was diagnosed with TB 1 year earlier. He had completed a 7-day course of amoxicillin and clavulanic acid on the recommendation of his pediatrician and he was afebrile only 1 day at the end of the antibiotic therapy. On clinical evaluation, his vital parameters were the following: temperature 39.4°C, heart rate 80 bpm, peripheral oxygen saturation 98%–99%, normal respiratory rate. Physical examination revealed right basal hypophonesis, with diffuse wheezing. Laboratory tests found an increased value of C-reactive protein (39.5 mg/L, n.v. 0.00–5.00 mg/L), normal white blood cell count (7.890/mmc, n.v 4.5–13.000/mmc) and neutrophils (6.050/mmc, n.v. 1.5–6.000/mmc), normal hepatorenal function.

Chest radiograph (Fig. 1C) showed a right hilar round-shaped parenchymal hypoventilation, surrounded by another medio-basal hypoventilation compatible with a tubercular excavation.

He was admitted to the pediatric ward and isolated according to the suspicion of active pulmonary tuberculosis. We performed Quantiferon test and microscopic, molecular and cultural diagnostic tests on 3 samples of sputum to detect *Mycobacterium tuberculosis* but all of them resulted negative. Bedside lung ultrasound showed 3 consolidated areas of parenchyma with air-arborized bronchograms and left basal pleural effusion.

Chest CT scan (Fig. 1D) performed to better characterize the formations described by radiograph and ultrasound revealed several bilateral areas of parenchymal consolidation, with irregular margins and a confluent appearance, and partially obstructed bronchi. Furthermore, the images showed areas with ground-glass appearance and several centrilobular micronodules, some of which had a tree-in-bud aspect. Also, in this case, there were several mediastinal lymphadenopathies, some of which were characterized by internal necrosis. Because of the high suspicion of tuberculosis, we prescribed first-line antitubercular treatment, interrupted 6 days later for intolerance. To obtain a definitive diagnosis, we required microbiologic analysis also on bronchoalveolar lavage fluid, which excluded a mycobacterium tuberculosis infection so we interrupted antitubercular treatment.

The main microbiologic finding was a nasopharyngeal swab positive for *Mycoplasma pneumoniae*. Since the general clinical conditions of the patient were improving and the fever disappeared 6 days after the admission, we did not prescribe a specific macrolide treatment and we repeated 1 month after the beginning of the clinical manifestations in outpatient setting a lung ultrasound which was normal.

*Mycoplasma pneumoniae* infection in children can cause different and noncharacteristic clinical, laboratory and radiologic manifestations.^7^ It is often responsible for self-limited community-acquired pneumonia, but sometimes it is the pathogen of severe low respiratory tract inflammation causing atelectasis and massive lung infiltration. In this article, we reported the cases of 2 children who came to our attention for persistent fever and with evidence of basal ipophonesis on respiratory auscultation. In both cases, CT scan showed parenchymal hypoventilation, areas with ground-glass appearance and centrilobular micronodules, some of which with a tree-in-bud aspect, compatible with the suspicion of tuberculosis. Interestingly both had severe mediastinal lymph node swelling. In a retrospective audit, we acknowledge the excessive diagnostic procedures (including invasive ones like CT and bronchoalveolar lavage), due to lack of awareness of this possible *Mycoplasma pneumoniae* localization and the social risk factors for TB.

Pediatric mediastinal lymphadenitis has been rarely reported in literature as a possible radiographic pattern of *Mycoplasma pneumoniae* infection. Lee et al^8^ analyzed the CT features of *Mycoplasma pneumoniae* pneumonia in 11 pediatric patients and they described hilar and/or mediastinal lymphadenopathies in 9 of them. In a Chinese prospective study published in 2016,^4^ the authors made an analysis of the diagnostic value of CT scan images in *Mycoplasma pneumoniae* pneumonia during the pediatric age. Among the 1280 children included in the study, 128 (corresponding to 10%) had hilar and/or mediastinal lymphadenitis. Hsieh et al^9^ described clinical and radiographic features in 39 children with *Mycoplasma pneumoniae* and they found lymphadenopathy in 5 children, corresponding to 13% of the population. The authors also looked for a relationship between lymphadenopathy, duration of hospitalization, C reactive protein level and patient age but they found no significative values. As van der Pijl et al^10^ suggested, *Mycoplasma pneumoniae* infection should be suspected also in adults with radiologic evidence of enlarged mediastinal lymph nodes.

These cases highlight the importance of considering *Mycoplasma pneumoniae* as a possible exclusive cause of mediastinal lymphadenitis, particularly if no other active tuberculosis clues like weight loss or positive interferon gamma release assays are associated. These reports are particularly important nowadays considering the huge increase in postpandemic *Mycoplasma pneumoniae* infections that is being recorded worldwide.^11^ As we learned from our experience, in these cases, close follow-up may be more reasonable rather than performing invasive diagnostic procedures. Our literature review showed that many authors focused only on the diagnostic phase, however it should be interesting to examine the clinical and radiologic evolution of these patterns as in our case the patients healed without a specific treatment in few weeks, therefore adding to the available knowledge about possible outcomes of *Mycoplasma pneumoniae*–associated mediastinal lymphadenitis.
